# Effect of physical therapy on bone remodelling in preterm infants: a multicenter randomized controlled clinical trial

**DOI:** 10.1186/s12887-022-03402-2

**Published:** 2022-06-24

**Authors:** Galaad Torró-Ferrero, Francisco Javier Fernández-Rego, María Rosario Jiménez-Liria, Juan Jose Agüera-Arenas, Jessica Piñero-Peñalver, María del Mar Sánchez-Joya, María Jesús Fernández-Berenguer, Mónica Rodríguez-Pérez, Antonia Gomez-Conesa

**Affiliations:** 1 International School of Doctorate of the University of Murcia (EIDUM), University of Murcia, 30100 Murcia, Spain; 2 Department of Physical Therapy, Faculty of Medicine University of Murcia, Espinardo 30100 Murcia, Spain; 3 Early Intervention Center of Lorca City Council, Lorca 30800 Murcia, Spain; 4 Pediatric Unit, Torrecárdenas University Hospital of Almería, Almería, Spain; 5 Neonatal Care Unit. Virgen de La Arrixaca Clinical University Hospital, Murcia, Spain; 6 Department of Developmental and Educational Psychology, University of Murcia, Murcia, Spain; 7 Faculty of Psicology, University of Murcia, Espinardo 30100 Murcia, Spain; 8 Department of Nursing, Physical Therapy and Medicine, University of Almería, Almería, Spain; 9 Neonatal Care Unit, General University Hospital of Elche, Alicante, Spain; 10 Physical Therapist, Torrecárdenas University Hospital of Almería, Almería, Spain; 11 Research Group Research Methods and Evaluation in Social Sciences. Mare Nostrum Campus of International Excellence, University of Murcia, Murcia, Spain

**Keywords:** Premature infant, Osteopenia, Physical therapy modalities, Neonatal intensive care units, Bone mineralization, Osteogenesis, Bone resorption

## Abstract

**Background:**

Preterm infants have a low level of bone mineralization compared to those born at term, since 80% of calcium incorporation occurs at the end of pregnancy. The purpose of the present study was to investigate the effect of reflex locomotion therapy on bone modeling and growth in preterm infants and to compare its effect with those of other Physiotherapy modalities.

**Methods:**

A multicentre randomized controlled clinical trial was conducted (02/2016 – 07/2020). 106 preterm infants born at the Virgen de la Arrixaca University Clinical Hospital, the General University Hospital of Elche and the Torrecárdenas University Hospital of Almería, between 26 and 34 weeks with hemodynamic stability, complete enteral nutrition and without any metabolic, congenital, genetic, neurological or respiratory disorders were evaluated for inclusion. Infants were randomly assigned to three groups: one group received reflex locomotion therapy (EGrlt); another group received passive mobilizations with gentle joint compression (EGpmc); and the control group received massage (CG). All treatments were carried out in the neonatal units lasting one month. The main outcome measure was bone formation and resorption measured with bone biomarkers. A mixed ANOVA was used to compare the results of bone biomarkers, and anthropometric measurements.

**Results:**

Infants were randomized to EGrlt (*n* = 38), EGpmc (*n* = 32), and CG (*n* = 36). All groups were similar in terms of gender (*p* = 0.891 female 47.2%), gestational age (M = 30.753, SD = 1.878, *p* = 0.39) and birth weight (M = 1413.45, SD = 347.36, *p* = 0.157). At the end of the study, significant differences were found between the groups in their interaction in bone formation, measured with osteocalcin [F (2,35) = 4.92, *p* = 0.013, η_p_^2^ = 0.043], in benefit of the EGrlt.

**Conclusions:**

Reflex locomotion therapy has been effective in improving bone formation, more so than other Physiotherapy modalities. Therefore, reflex locomotion therapy could be considered one of the most effective physiotherapeutic modalities for the prevention and treatment of osteopenia of prematurity.

**Trial registrstion:**

Trial retrospectively registered at ClinicalTrials.gov. First posted on 22/04/2020. Registration number: NCT04356807.

**Supplementary Information:**

The online version contains supplementary material available at 10.1186/s12887-022-03402-2.

## Background

Osteopenia of prematurity is a multifactorial pathogenic entity, of progressive course, that presents a variable severity and is characterized by bone demineralization [1].

Bone is a dynamic tissue in continuous resorption and formation, involving the formation of new bone, mediated by osteoblasts, and the resorption of old bone, which is carried out by osteoclasts. The amount of bone mass depends on the balance between these two activities, that is, it depends on the rate of bone turnover [2]. In normal homeostasis, bone metabolism is in balance to maintain the mass and microstructure of the skeleton.

The frequency with which the bone surface is activated will determine the number of remodeling zones present in the bone, so the sum of all the remodeling activity throughout the skeleton can be evaluated by measuring the biochemical markers of the bone remodeling in serum or urine [3]. In contrast, bone mass measurements and radiographs provide a static picture of a specific skeletal region [4].

In this sense, biochemical markers of bone resorption and formation can provide an idea of the mechanical effects of tactile and kinesthetic stimulation on bone development; and although serum is difficult to collect from preterm infants, many biomarkers can also be detected in urine, which is easier to obtain. Furthermore, in a study on postnatal bone mineralization, they concluded that the osteopenia observed in preterm infants is caused by increased bone resorption, measured in urine, and not by decreased bone formation [5].

For this reason, it is important to remember that the resorption process is faster than the formation process, and consequently any increase in the remodeling cycle leads to a loss of bone mass. Thus, in theory, if the bone remodeling cycle is coupled, both an increase in a resorption marker or a decrease in a formation marker could predict future loss of bone mass.

In this sense, the most specific and sensitive resorption markers are the N-telopeptides of the collagen bonds (NTx) and the C-telopeptides of the collagen bonds (Beta-CTx). Likewise, its increased levels, regardless of bone mineral density, are predictors of fracture risk [6]. Regarding the formation markers, the most sensitive are bone-specific alkaline phosphatase (BSAP), osteocalcin (OC), and carboxyl-terminal collagen type I propeptides (PINP) [2].

In relation to preterm infants, mineralization is much lower than the expected intrauterine bone mineralization, clinical characteristics are nonspecific and often appear as a late symptom [5]. In addition, these poor mineralization rates are maintained in children and young adults born prematurely [7], a situation that leads, in the long term, to a reduction in maximum bone mass, weaker bones, shorter stature, and an increased risk of fracture compared with those born at term [8].

For the treatment and prevention of osteopenia in preterm infants, physiotherapy modalities have shown favourable results when applying passive movements with gentle compression [9], based on the fact that mechanical loading on bones and joints stimulates bone formation and growth [10]. Absence of mechanical loading, as seen in spaceflight and bedridden adults, increases bone resorption and hypercalcuria and decreases bone mass [11].

In this sense, it has been shown that mechanical stress is one of the most stimulating factors of bone formation and growth, increasing bone mass in children, adolescents and adults [12].

Based on these findings, reflex locomotion therapy (RLT) [13] may be an appropriate method to generate involuntary activity, developing active-resistive movement in the population of preterm infants.

RLT consists in the activation of innate locomotion patterns, through proprioceptive stimuli that produce a response from the central nervous system (CNS). To trigger these patterns, we must place the baby in a certain posture and apply finger pressure to specific areas or points [14–16] to generate proprioceptive stimuli of the periosteal and muscle stretching type. The response to these stimuli consists of a series of synergistic muscular contractions, which involve the whole body, and which trigger specific, active and involuntary movement patterns in the child [14–16].

To date, this therapeutic modality in premature infants has only shown its efficacy in relation to the improvement of respiratory parameters [13, 14, 17, 18], and no stress or pain has been observed in this population [13]. Even so, observing the effects on the musculoskeletal system and on the central nervous system, already demonstrated in adults as mentioned above, this therapeutic modality could improve bone remodeling in premature infants.

## Methods

### Study design

The aim of the present study was to verify preterm infants’ improvement in bone formation and resorption in response to a physiotherapy intervention program with RLT, and to compare it with another physiotherapeutic procedure that has proven to be effective in the treatment of osteopenia in the preterm infant.

A multicenter randomized prospective clinical trial, carried out in the neonatology services of the Virgen de la Arrixaca Clinical University Hospital (HUCVA), the Torrecárdenas University Hospital of Almería (HTA) and the General University Hospital of Elche (HGUE); from February 2016 to July 2020. This trial included 106 preterm infants divided into three intervention groups: two treatment and one control. This study was approved by the HUCVA, HGUE and HTA clinical research ethics committee and all the procedures stipulated in the Helsinki declaration were carried out [19]. Likewise, the CONSORT recommendations were followed for the preparation and writing of randomized clinical trial [20]. This study has been registered with ClinicalTrials.gov with identification number: NCT04356807.

### Subjects

Preterm infants admitted to the neonatal units of the HUCVA, HGUE and HTA hospitals, and born between 26 to 34 weeks of gestational age, hemodynamically stable and with complete enteral nutrition, whose parents or guardians signed an informed consent authorizing the participation of the baby in this study.

Babies presenting neurological disorders, mechanical ventilation, bronchopulmonary dysplasia, congenital malformations, metabolic diseases, genetic diseases, grade 3–4 intraventricular hemorrhage, as well as those who were taking diuretic or corticosteroid medication, and those who had bone fractures at the time of inclusion, were excluded.

The practices regarding feeding protocol were standardized between hospitals, considering the enteral nutrition as 180 ml/kg/day, giving priority to human milk (no differences found between groups regarding the type of milk intake), and oral vitamin D intake was started at 14 days of life at a rate of 800 IU per day, except for those who have received parenteral nutrition, in which case started at 48 h since vitamins are included in parenteral nutrition.

### Interventions

The participants in this study were divided into three groups, which received different Physiotherapy treatments, along with the standard nursing care.

Control group (CG), was given limb and core massage, with gentle deep pressures and caresses; lasting 15 min a day in a single Physiotherapy session, 5 days per week, for 4 weeks; considering it a placebo since this intervention has no influence on bone mineralization [21–26].

Experimental group (EGpmc), with passive movements with gentle joint compression (PMC), described by Moyer-Mileur, et al. [27] and with the adaptations of Vignochi, et al. [28] in a 15 min Physiotherapy session, 5 days per week for 4 weeks. These mobilizations consist of flexion and extension movements in all the joints of both the upper and lower extremities and ending with chest movements following the baby's respiratory pace.

Experimental group (EGrlt), with RLT according to the procedures used by other authors [13–15], for 16 min divided into two Physiotherapy sessions of 8 min each, 5 days per week for 4 weeks. The exercises corresponding to the motor complexes of the 1st phase of reflex rolling and the reflex creeping were performed, spending one minute on each side and performing two repetitions per session.

For the 1st phase of reflex rolling, the child is placed in dorsal decubitus, with the head turned to one side at an angle of 30º, the spine as aligned as possible, and limbs relaxed. The physiotherapist makes gentle pressure with his thumb, at the point of intersection of the mammillary line with the diaphragm, between the 6th-7th intercostal space, in the hemithorax on the side towards which the head rotates, with a dorsal-medial-cranial direction, while resisting with the other hand the turning of the head towards the other side [13, 14, 16].

For reflex creeping, the child is placed proned, passively bringing the head to axial neck extension at 30 degrees of rotation. The upper limb, on the side to which the head is turned, is placed in a position of shoulder flexion between 120 and 135 degrees, with 30 degrees of abduction, leaving the epitrochlea supported; the wrist is aligned with the shoulder, the forearm rests on the palmar face, and the longitudinal axis of the humerus points towards the vertex of the lumbosacral hinge. The opposite arm is placed relaxed parallel towards the longitudinal axis of the body.

The leg on the side to which the child's head is turned to, rests extended and relaxed. The other leg is placed with the hip in external rotation and abduction, leaving the support on the internal condyle of the femur, the knee slightly flexed, and the heel aligned with the ischium. The stimulation is carried out, with the index finger of one hand, on the lateral tuberosity of the calcaneus, in the ventral-cranial-medial direction of the leg opposite to the side to which the head is turned, and with the index finger of the other hand, on the epitrochlea of the arm towards which the head is turned, in a dorsal-medial-cranial direction [15].

One physiotherapist (always the same) per hospital, in all cases with more than five years of experience, was in charge of performing the treatments. The participants of the three groups were treated in the incubator or crib and evaluated under the same conditions.

All participants were assessed with the Neonatal Infant Pains Scale (NIPS) [29–31] by experimented nursing staff during the procedures, this scale is assessed in all hospitals by protocol whenever an intervention is to be performed on neonates. The NIPS scale considers an intervention as not painful or not stressful if a score of 0–2 is obtained, moderately stressful or painful if a score of 3–4 is obtained, or very stressful or intense pain if the score is greater than 4 [31, 32]. In addition, also by protocol, all neonates were given between 0.5 ml and 1 ml of a 24% oral sucrose solution 2 min before the start of the intervention [33, 34].

### Outcomes

In this trial, we have studied bone formation, bone resorption, and anthropometric measurements of weight, height, and head circumference.

In order to measure bone formation and resorption, we have used serum and urine biomarkers, respectively, to reveal analytical data on the rate of bone formation and its metabolism [35]. Specifically, in bone formation we use OC markers, as it is one of the most sensitive markers of bone formation [2]. Among the existing resorption biomarkers, we specifically use the N-Tx in urine (NTx-urine), the NTx in serum (NTx-serum) and the Beta-CTx, since they are the most sensitive and specific markers for the measurement of this quality [2, 6].

In order to analyze the anthropometry, measurements of weight, height and head circumference were collected. Body weight was measured with a digital scale without clothing, height was measured as the distance from head to heel with a non-elastic tape, and the head circumference as the cephalic contour at its widest part, between the eyebrows and the occiput, also measured with a non-elastic band.

Urine biomarker tests were performed one day before starting the sessions, two weeks after, and at the end of treatment. Serum biomarkers were performed only one day before starting the sessions and once again at the end of the treatment. Serum biomarkers, during the hospitalization period, were always taken according to blood collection guidelines already ordered by their doctor. For ethical reasons, in no case were blood samples taken exclusively for the purposes of this study. The extraction was carried out by the nursing staff, who were masked for the purposes of the present study and did not know which group the participants belonged to. Anthropometric measures were taken from one day before the start of the treatment to one day after finishing it, collecting them in alternating days, in agreement with the nursing protocol, and were carried out by said staff. For our analysis, we used those that coincided with the day the urine was collected, and failing that, the last measurement performed before that day.

The Z score was calculated for birth weight and for weight, height and head circumference at the different measurement moments, following the 2013 Fenton growth charts for this purpose [36, 37].

### Sample size

We calculated the sample size using the f^2^ statistic with Cohen's criteria, using an f^2^ value between moderate and low of 0.15, which is between 0.10 (Low) and 0.25 (moderate). We assume a significance level of 5% and statistical power of 80% with three levels for each of the intra and inter group factors. In this way, a total sample size of 93 participants was projected, at a rate of 31 patients per group. The statistical program used was G*Power 3.1.9.2 [38]. We use this method because there are no previous studies with 3 groups [39].

Assuming possible deviations from these premises, we opted for a sample of 33 patients per group. As the present study requires three treatment groups, the final sample size was was of 99 participants, who, distributed among the three hospitals, would give us a sample of 33 babies per hospital. Each hospital would then have an equal number of 11 patients per treatment group.

### Randomisation

The groups were formed by simple randomization. The randomization procedure was the same for each hospital and consisted of mixed labels within a sealed, opaque envelope. These labels contained an assignment number for each group. Each time a new participant was proposed, a person outside the research randomly drew a number and performed the assignment. For ethical reasons, identical twins, non-identical twins, and triplets were assigned to the same group.

### Blinding

All the personnel who carried out the measurement tests are external to the study and were blinded to the intervention group to which the patients belonged. Likewise, participants, family, and data analysts were also blinded. The physiotherapists who carried out the treatments were blinded against the objectives of the study, they did not know which were the main study variables, nor which were the experimental and control treatments.

### Statistical methods

The computerized statistical package "R", version 4.0.3 (R Core Team 2020) [40] was used to perform all data analyzes.

The participants’ qualitative baseline gender characteristics were compared using a contingency table and a Chi square test was performed for their analysis; while for the characteristics of gestational age at birth, weight at birth, birth weight Z score, gestational age at the beginning of the intervention, and weight, height, head circumference, anthropometric Z scores and urine and serum biomarkers a one-factor analysis of variance was performed. On the other hand, a mixed analysis of variance (ANOVA) was carried out to compare the effect of the intervention on anthropometric measures of weight, height and head circumference, anthropometric Z scores and for urine and serum biomarkers. In those cases where the homoscedasticity assumption was not met, a robust mixed ANOVA was carried out. Bonferroni was used to adjust the 95% confidence intervals (95%CI).

An intention-to-treat statistical analysis was performed. Statistical significance was stipulated with a p < 0.05. For the effect size, the eta squared (η^2^) was calculated, which will be considered high when its value is > 0.14, moderate when its value is between 0.14 and 0.06 and small when its value is between 0.06 and 0.01 [41]. Data are presented as mean ± standard deviation.

## Results

### Participants

**Fig. 1:**
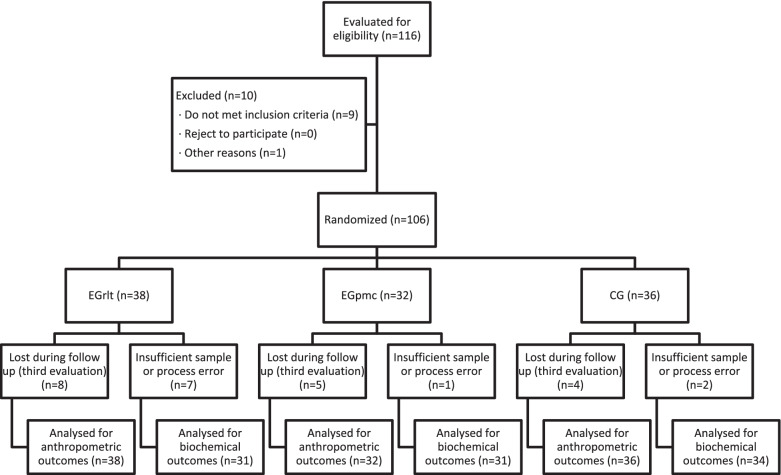
Participants flow diagram

116 preterm infants admitted to the three hospitals (52 preterm infants admitted to HUCVA; 25 in the HGUE; and 39 in the HTA) were selected to be included in the study from February 2016 to July 2020 (Fig. 1). From these, 10 were ruled out due to exclusion criteria prior to achieving complete enteral nutrition or at the time of randomization. In the HUCVA, 6 patients were ruled out: one of them developed grade IV intraventricular hemorrhage, two developed necrotizing enterocolitis that required surgical intervention, and three of them were on mechanical ventilation at the time of acquiring full enteral nutrition. In the HGUE, 3 patients were ruled out: one due to exitus, another due to hospital transfer and the last due to generating a grade III intraventricular hemorrhage. In HTA, one was ruled out due to necrotizing enterocolitis. Thus, the total number of participants resulted in 106 patients, 46 in the HUCVA, 22 in the HGUE and 38 in the HT. After randomization, 38 were assigned to EGrlt, 32 to EGpmc, and 36 to CG.

The last measurement could not be performed in 17 of the 106 preterm infants who started the study, as they were discharged before the end of the 4 weeks of treatment: 11 from HUCVA (4 from EGrlt, 4 from EGpmc and 3 from CG), 1 from HGUE (EGrlt) and 5 from HTA (3 from EGrlt, 1 from EGpmc and 1 from CG). Since the statistical analysis was done by intention-to-treat, the values of the last measurement taken were used for the final measurement in those cases where these data were missing.

The three groups’ participants’ characteristics were similar in gender (*p* = 0.891, female 47.2%), gestational age (M = 30.753, SD = 1.878, *p* = 0.39), birth weight (M = 1413.45, SD = 347.36, *p* = 0.157) and birth weight Z score (M = -0.27; SD = 0.84; *p* = 0.469), thus forming a group of large preterm infants with very low birth weight. Neither were there differences in gestational age at the beginning of the intervention (M = 33.562, SD = 1.444, *p* = 0.891) height (M = 42.018, SD = 2.164, *p* = 0.052) nor head circumference (M = 28.471, SD = 3.998, *p* = 0.147) at the time of initiating the intervention. The groups were similar in terms of NTx-urine (M = 4756.665, SD = 2124.473, *p* = 0.164), NTx-serum (M = 472.65, SD = 103.057, *p* = 0.598), Beta-CTx (M = 0.874, SD = 0.284, *p* = 0.864) resorption biomarkers; and OC formation biomarker (M = 72.032, SD = 31.4, *p* = 0.614) at the beginning of the intervention.

In contrast, significant differences were found in terms of weight (M = 1655.076, SD = 277.63, *p* = 0.022) at the time of initiating the interventions; the weight data of the EGpmc being higher than the EGrlt (Table 1).

**Table 1:** Participants characteristics

	**EGrlt (*n* = 38)**	**EGpmc (*n* = 32)**	**CG (*n* = 36)**
Gestational age (weeks)	30.44 ± 1.96	31.04 ± 1.82	30.82 ± 1.83
Birth weight (g)	1350.39 ± 352.92	1503.88 ± 373.85	1399.64 ± 307.44
Birth weight Z score	-0.31 ± 0.78	-0.14 ± 1.01	-0.34 ± 0.74
Gender (female)	19 (50%)	15 (46.9%)	16 (44.4%)
Gestational age at the beginning of the intervention (weeks)	33.48 ± 1.53	33.56 ± 1.54	33.65 ± 1.29
Weight at the beginning of the intervention (g)*	1611.47 ± 202.75	1768.22 ± 387.24*	1600.53 ± 195.27
Height at the beginning of the intervention (cm)	41.38 ± 1.67	42.77 ± 2.83	42.1 ± 1.8
Head circumference at the beginning of the intervention (cm)	27.5 ± 4.44	29.46 ± 3.99	28.46 ± 3.78
NTx-urine	4266.11 ± 1441.48	5409.33 ± 2724.29	4604.38 ± 1918.98
NTx-serum	457.01 ± 109.59	473.38 ± 95.84	487.71 ± 107.05
Beta-CTx	0.79 ± 0.21	0.95 ± 0.31	0.91 ± 0.32
OC	61.56 ± 28.11	78.1 ± 34.44	78.18 ± 31.51

^*^p < 0.05 for differences between groups. *CG*  Control Group, *EGpmc*  Experimental group treated with passive mobilizations with gentle joint compression, *EGrlt*  Experimental group treated with reflex locomotion therapy. *NTx* N-Telopeptides of collagen bonds, *Beta-CTx* C-telopeptides of collagen bonds, *OC* Osteocalcin. Data is presented as Mean ± SD

### Intervention effect on bone biomarkers

When performing the biochemical analysis, there were 4 cases in which the blood and urine samples were too small to perform the tests with the biochemical reagents and 6 cases in which the test failed and the samples were lost. Therefore, the sample analyzed for the biochemical variables was EGrlt = 31, EGpmc = 31, CG = 34.

#### Bone resorption biomarkers

##### NTx-urine

**Fig. 2:**
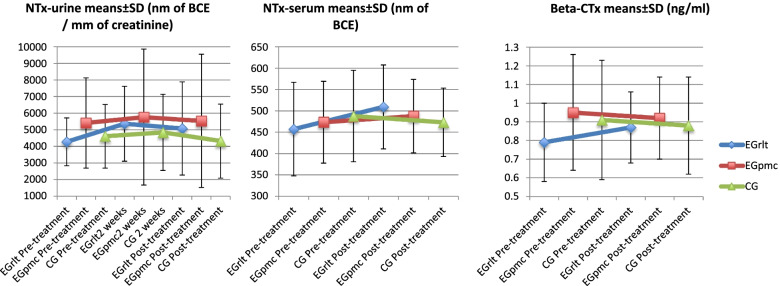
Interaction of the bone resorption outcomes in the different groups. Legend: EGrlt = Experimental group treated with reflex locomotion therapy. EGpmc = Experimental group treated with passive mobilizations with gentle joint compression. CG = Control Group. NTx: N-Telopeptides of collagen bonds. Beta-CTx: C-telopeptides of collagen bonds. nm: nanometres. mm: millimetres. BCE: Bone collagen equivalents. ng: nanogram. ml: Millilitres. Data is presented as Mean ± SD

Regarding the NTx-urine biomarkers, when making the comparison with the group, we observe no significant differences in terms of the values obtained at the different measurement times [F (2,184) = 1.429, p = 0.242, η^2^ = 0.007], the values obtained by the different groups [F (2,92) = 1.936, p = 0.15, η^2^ = 0.022] and in the interaction of the variable in the different groups [F (4,184) = 0.547, p = 0.701, η^2^ = 0.005] (Fig. 2, Table 2).

**Table 2:** Effect of the different interventions in the biochemical outcomes

		EGrlt (*n* = 31)	EGpmc (*n* = 31)	CG (*n* = 34)	Pairwise comparation	Mean differences	CI95%	
							Lower bound	Upper bound
NTx-urine (nm of BCE / mm of creatinine)	Pre-treatment	4266.11 ± 1441.48	5409.33 ± 2724.29	4604.38 ± 1918.98	EGrlt = EGpmc	-1143.2	-2435.8	149.36
					EGrlt = CG	-338.3	-1611.1	934.58
					EGpmc = CG	805	-467.9	2077.8
	2 weeks	5362.30 ± 2257.88	5759.57 ± 4094.29	4835.02 ± 2290.11	EGrlt = EGpmc	-397.3	-2245.6	1451.1
					EGrlt = CG	527.3	-1292.81	2347.4
					EGpmc = CG	924.5	-895.5	2744.6
	Post-treatment	5070.38 ± 2807.67	5529.16 ± 4016.74	4317.45 ± 2231.91	EGrlt = EGpmc	-458.8	-2369.2	1451.6
					EGrlt = CG	752.9	-1128.3	2634.2
					EGpmc = CG	1211.7	-669.5	3092.9
NTx-serum (nm of BCE)	Pre-treatment	457.01 ± 109.59	473.38 ± 95.84	487.71 ± 107.05	EGrlt = EGpmc	-16.37	-122.31	89.6
					EGrlt = CG	-30.7	-130.08	68.7
					EGpmc = CG	-14.33	-120.27	91.61
	Post-treatment	509.53 ± 98.3	494.63 ± 85.96	473.09 ± 80.11	EGrlt = EGpmc	14.9	-74.54	104.34
					EGrlt = CG	36.44	-47.46	120.34
					EGpmc = CG	21.54	-67.9	111
Beta-CTx (ng/ml)	Pre-treatment	0.79 ± 0.21	0.95 ± 0.31	0.91 ± 0.32	EGrlt = EGpmc	-0.16	-0.45	0.13
					EGrlt = CG	-0.122	-0.4	0.15
					EGpmc = CG	0.038	-0.25	0.33
	Post-treatment	0.87 ± 0.19	0.94 ± 0.22	0.88 ± 0.26	EGrlt = EGpmc	-0.065	-0.29	0.16
					EGrlt = CG	-0.005	-0.22	0.21
					EGpmc = CG	0.059	-0.17	0.29
OC (ng/ml)	Pre-treatment	61.56 ± 28.11	78.1 ± 34.44	78.18 ± 31.51	EGrlt = EGpmc	-16.543	-48.1	14.97
					EGrlt = CG	-16.62	-46.74	13.5
					EGpmc = CG	-0.077	-32.12	31.97
	Post-treatment	99.74 ± 34.05	97.35 ± 36.44	86.12 ± 24.52	EGrlt = EGpmc	2.397	-29.8	34.6
					EGrlt = CG	13.62	-17.16	44.4
					EGpmc = CG	11.222	-21.52	43.96

^*^p < 0.05 for differences between groups. *CG*  Control Group, *EGpmc*  Experimental group treated with passive mobilizations with gentle joint compression, *EGrlt*  Experimental group treated with reflex locomotion therapy, *CI95%*  95% Confidence Interval, *NTx* N-Telopeptides of collagen bonds. *Beta-CTx*: C-telopeptides of collagen bonds, *OC* Osteocalcin, *nm* Nanometres, *mm* Millimetres, *BCE* Bone collagen equivalents, *ng* Nanogram, *ml* Millilitres. Data is presented as Mean ± SD

##### NTx-serum

In the case of the NTx-serum biomarkers, when making the comparison with the group, we also observe that there are no significant differences in terms of the values obtained at the different measurement times [F (1,36) = 2.277, *p* = 0.14, η^2^ = 0.011], the values obtained by the different groups [F (2,36) = 0.006, *p* = 0.993, η^2^ < 0.001] and the interaction of the variable in the different groups [F (2,36) = 2.404, *p* = 0.105, η^2^ = 0.023] (Fig. 2, Table 2).

##### Beta-CTx

Regarding the Beta-CTx biomarkers, once again we observe no differences in terms of the values obtained at the different measurement times [F (1,35) = 0.121, *p* = 0.73, η^2^ < 0.001], the values obtained by the different groups [F (2,35) = 0.852, *p* = 0.435, η^2^ = 0.034] and the interaction of the variable in the different groups [F (2,35) = 0.712, *p* = 0.497, η^2^ = 0.011] (Fig. 2, Table 2).

#### Bone formation biomarkers

##### OC

In the OC biomarkers, when making the comparison with the group, we observe that there are significant differences in terms of the values obtained at the different times of measurement [F (1,35) = 27.84, *p* < 0.001, η^2^ = 0.114], and the interaction of the variable in the different groups [F (2,35) = 4.92, *p* = 0.013, η^2^ = 0.043]; but not in the values obtained by the different groups [F (2,35) = 0.198, *p* = 0.821, η^2^ = 0.009].

On the one hand, we observe that between the first and the last measurement, the EGrlt (*p* < 0.001; 95% CI = 24.44 to 51.93) and the EGpmc (*p* = 0.016; 95% CI = 3.74 to 34.75) significantly evolve, but not the CG (*p* = 0.266; 95% CI = -6.32 to 22.21).

**Fig. 3:**
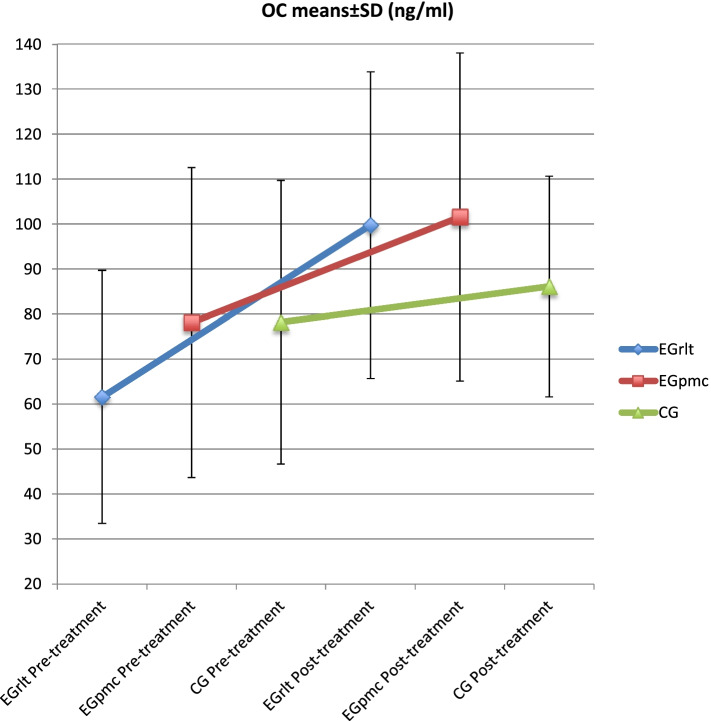
OC interaction regarding the group. Legend: EGrlt = Experimental group treated with reflex locomotion therapy. EGpmc = Experimental group treated with passive mobilizations with gentle joint compression. CG = Control Group. OC: Osteocalcin. ng: nanogram. ml: Millilitres. Data is presented as Mean ± SD

On the other hand, the results show that there are significant differences between the groups, regarding how they evolve at the different times of OC measurement, as shown in the Fig. 3, we can see that the group with a better evolution is the EGrlt, and the group with the worst evolution is the CG (Fig. 3, Table 2).

### Intervention effect on anthropometric outcomes

#### Weight

When comparing the weight with that of the group, we observe that there are significant differences in terms of the values obtained at the different measuring times [F (2,116) = 512.772, *p* < 0.001, η^2^ = 0.548, observed power = 0.64], and the group [F (2,58) = 4.245, *p* = 0.019, η^2^ = 0.112]; but not in the interaction [F (4,116) = 2.351, *p* = 0.078, η^2^ = 0.011, observed power = 0.64].

**Fig. 4:**
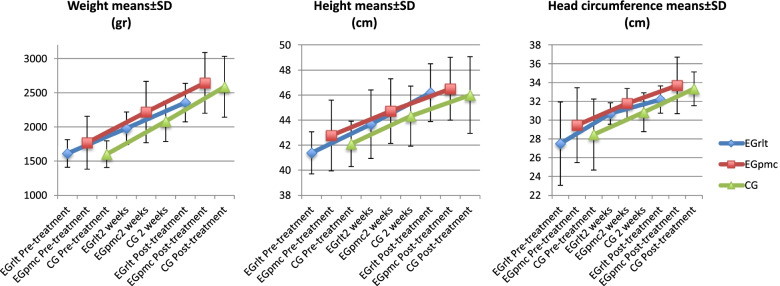
Interaction of the anthropometric outcomes in the different groups. Legend: EGrlt = Experimental group treated with reflex locomotion therapy. EGpmc = Experimental group treated with passive mobilizations with gentle joint compression. CG = Control Group. Data is presented as Mean ± SD

Thus, when making the pairwise comparison between the groups and the different measuring times, the results show a sustained significant difference between the EGtrl and the EGpmc in favor of the latter in each of the measures (Table 3), but since no differences are found in the interaction, we can state that all groups evolved similarly (Fig. 4).

**Table 3:** Effect of the different interventions in the anthropometric outcomes

		EGrlt (*n* = 38)	EGpmc (*n* = 32)	CG (*n* = 36)	Pairwise comparation	Mean differences	CI95%	
							Lower bound	Upper bound
Weight (g)	Pre-treatment	1611.47 ± 202.74	1768.22 ± 387.25	1600.53 ± 195.27	EGrlt < EGpmc*	-230.65	-408.96	-52.35
					EGrlt = CG	-71.84	-200.55	56.86
					EGpmc = CG	158.81	-31.84	349.46
	2 weeks	1979.69 ± 239.06	2218.26 ± 450.18	2082.4 ± 295.36	EGrlt < EGpmc*	-304.7	-541.86	-67.51
					EGrlt = CG	-119.4	-288.54	49.8
					EGpmc = CG	185.3	-69.85	440.49
	Post-treatment	2356.0 ± 281.36	2644.46 ± 444.24	2587.37 ± 444.65	EGrlt < EGpmc*	-329.55	-561	-98.09
					EGrlt = CG	-270.53	-502.7	-38.4
					EGpmc = CG	59.02	-222.9	340.9
Height (cm)	Pre-treatment	41.38 ± 1.68	42.77 ± 2.83	42.1 ± 1.81	EGrlt = EGpmc	-3	-5	-1
					EGrlt = CG	-1	-2	1
					EGpmc = CG	2	0	4
	2 weeks	43.67 ± 2.74	44.72 ± 2.58	44.32 ± 2.4	EGrlt = EGpmc	-1.471	-3.276	0.335
					EGrlt = CG	-0.092	-2.13	1.946
					EGpmc = CG	1.379	-0.34	3.097
	Post-treatment	46.19 ± 2.3	46.5 ± 2.5	46.0 ± 3.06	EGrlt = EGpmc	-0.559	-2.218	1.1
					EGrlt = CG	-0.029	-1.899	1.84
					EGpmc = CG	0.529	-1.471	2.53
Head circumference (cm)	Pre-treatment	27.5 ± 4.44	29.46 ± 3.99	28.46 ± 3.78	EGrlt < EGpmc**	-5.052	-9.443	-0.661
					EGrlt = CG	-3.052	-7.439	1.335
					EGpmc = CG	2	-1.665	5.665
	2 weeks	30.7 ± 1.14	31.77 ± 1.6	30.84 ± 2.07	EGrlt = EGpmc EGrlt = CG EGpmc = CG	-1.219 -0.718 0.5	-2.525 -2.117 -0.821	0.087 0.679 1.821
	Post-treatment	32.19 ± 1.45	33.69 ± 3.0	33.34 ± 1.8	EGrlt = EGpmc EGrlt = CG EGpmc = CG	-1.448 -1.135 0.313	-3.467 -2.406 -1.56	0.572 0.135 2.185

^*^p < 0.05 for differences between groups

**pseudosignificant differences. *CG*  Control Group, *EGpmc*  Experimental group treated with passive mobilizations with gentle joint compression, *EGrlt*  Experimental group treated with reflex locomotion therapy, *CI95% *  95% Confidence Interval. Data is presented as Mean ± SD

In contrast, all the groups’ weight measurements evolved significantly in each of their weight measurements, compared to the previous time measured.

#### Height

When comparing the height with respect to the group, we observe that there are significant differences in terms of the values obtained at the different measuring times [F (2,23.547) = 65.606, *p* < 0.001, η^2^ = 0.339], and in the interaction of the variable in the different groups [F (4,19.384) = 3.855, *p* = 0.018, η^2^ = 0.027]; but not in the values obtained by the different groups [F (2,19.651) = 1.445, *p* = 0.26, η^2^ = 0.339].

As with the OC variable, there are significant differences in terms of interaction, so we must look at Fig. 4 to see that the group presenting a better evolution with respect to the other groups,is the EGrlt, while CG is the one with the worst evolution.

Just as with the weight, all groups increased their height significantly at the different measuring times, compared to their previous measurement.

#### Head circumference

When comparing the head circumference with the group, we observe significant differences in terms of the values obtained at the different measuring times [F (2,82) = 37.904, *p* < 0.001, η^2^ = 0.38, observed power = 0.285], and the group [F (2,41) = 5.375, *p* = 0.008, η^2^ = 0.081]; but not regarding the interaction [F (4,82) = 1.276, *p* = 0.291, η^2^ = 0.04, observed power = 0.285].

When doing the pairwise comparison, the EGpmc starts from position of advantage, close to significant, with respect to the EGrlt (*p* = 0.056); this situation no longer occurs throughout the remaining measurements between any of the groups (Table 3). Furthermore, since there are no differences in terms of interaction, we can say that all groups evolve equally in their increase of head circumference (Fig. 4).

Finally, regarding the separate evolution of the groups, they all evolve significantly in their respective measurements compared to their previous ones.

Regarding the Z scores, no differences are observed in terms of the interaction of these variables on the group with respect to weight [F (4,204) = 0.995, *p* = 0.399, η^2^ = 0.019], height [F (4,204) = 1.037, *p* = 0.361, η^2^ = 0.02] and head circumference [F (4,204) = 0.477, *p* = 0.635, η^2^ = 0.009].

### Harms

No adverse effects were detected in the participants as a consequence of the different interventions. Any participant showed any signs of stress or pain during the interventions measured with NIPS. Neither participant developed any fracture or adverse pathology such as intraventricular hemorrhage.

## Discussion

### Limitations

One of the limitations that we can extract from our study is the lack of long-term follow-up of the main variables, to determine if the effect of the intervention is maintained over time. Another limitation is related to the heterogeneity of the preterm population in terms of their risk factors, since this fact could influence the results obtained.

### Generalisability

Due to the characteristics of the sample on which the intervention has been carried out, the results indicate that treatment with RLT has a positive effect, increasing bone formation in healthy preterm infants. For that reason, it could be considered one of the best treatments for the improvement of bone remodeling in this population.

### Interpretation

The aim of this research was to verify the effect of RLT, understood as active-resistive mobilizations, on bone formation, bone resorption and growth in preterm infants, and to compare its effect with other passive Physiotherapy modalities.

Firstly, it should be noted that although we initially considered a sample of 99 premature babies distributed in groups of 33 participants per hospital, in the end, the sample consisted of 106 participants, 46 from HUCVA, 22 from HGUE and 38 from HTA. This difference is due to the lack of availability of participants in the HGUE, thus in order to complete the study within the stipulated deadlines, it was agreed that the HUCVA (with greater availability) would increase its sample to compensate that of the HGUE.

After the results’ analysis, we observed that RLT has a significant positive effect, with low effect size, on bone formation and bone growth in preterm infants, observed in OC bone formation biomarkers and height; which may have a positive effect on osteopenia in this population.

In this sense, if we focus on the resorption variables, we can state that the intervention model has no effect on bone resorption, although, in appearance, if we observe the interaction graphs (Fig. 3), the group with the highest resorption is the Egrlt. This fact can be considered positive, since we know that resorption is necessary to increase bone formation and to have correct bone remodeling [42]. This aspect is related to bone formation data, since EGrlt is the group that best evolves with respect to the other groups.

These results are in agreement with those found by Sezer Efe, et al. [43], who do not find significant differences between the control group and the intervention group to which they applied PMC in their bone formation variables. Other authors find differences in their training variables between the control group and the group treated with PMC [22, 24, 25, 44], but the biomarkers they use in their studies are not the most sensitive and the sample used in their trials is smaller than the one in this study. Currently, the most sensitive biomarkers of bone formation are OC biomarkers, carboxy-terminal propeptides, and bone-specific alkaline phosphatase biomarkers [2]. Nevertheless, for this study, we did not use carboxyterminal propeptide biomarkers because type I collagen also appears in tissues other than bone, and this limits its use in the study of metabolic and bone pathology [45]. Neither did we use those of bone alkaline phosphatase, since when performing this test in preterm infants, the placental isoenzyme may show an error in its values [46].

Furthermore, our results are also in line with those of other authors who observe the effect of PMC on bone resorption. El-Farrash, et al. [47] found no differences in favor of the group treated with PMC in the variable of Beta-CTx; in fact, only 3 [21, 26, 48] of the studies found that used the resorption variables found significant differences in favor of the group treated with PMC. Nevertheless, these authors use ICTP and Dpd measurements, and we currently know that the most sensitive biomarkers for the detection of changes in resorption are the telopeptide forms Beta-CTx in serum and NTx in urine [6].

In the CG, the results show, as we expected, that massage and tactile stimulation has no effect on bone remodeling. In this sense, other authors reach the same conclusions [22, 25, 49].

Regarding the anthropometric variables, it could be thought that since the RLT is a treatment where infants perform an active-resistive exercise, this could produce an adverse effect in terms of weight gain compared to passive therapies; however, the intervention does not appear to have an effect on weight. Even though the group that best evolves is the CG treated with massage as a placebo, and although the positive effect of massage on weight gain in preterm infants has already been observed [50], it does not evolve in a significant way compared to the other groups; meaning that they all evolve similarly. Thus, the intervention modality does not appear to have an effect on weight.

In the other anthropometric variables, we observe that the RLT has a positive and significant effect on height. Furthermore, this effect was not observed in the group treated with PMC compared to CG treated with massage, as has been shown by other authors who did not observe this difference either [24, 43].

In the head circumference variable, significant differences are found between the groups, but later, when doing the Post-hoc and the pairwise comparison, this no longer occurs. This is due to the fact that a Bonferroni correction is made; consisting of a very strong correction avoiding type II error, which is why these differences are no longer observed and only a difference is found close to a statistical significance, with a moderate effect size, between EGpmc and EGrlt at the start of the intervention in favor of the first group. These differences diminish in the following evaluations, so that we may say that the EGrlt’s evolution is more favorable, even if not significantly so, since it started from a position of disadvantage.

Finally, regarding stress or pain, these results are in agreement with those obtained by Giannantonio, et al. 2010 [13], who assessed NIPS and Preterm Infant Pain Profile and whose results remained unmodified regarding these scales during the reflex locomotion therapy.

## Conclusions

We may conclude that RLT is an effective treatment for increasing bone formation and growth in preterm infants. This fact may have a positive effect on the prevention of osteopenia in this population. Furthermore, RLT has been shown to be more effective than other Physical Therapy modalities such as PMC and massage in improving bone formation and growth.

It would be advisable to carry out long-term follow-up studies, where the evolution of these children in early childhood and adolescence would be observed.

## Supplementary Information

**Additional file 1: Table S1.** Raw Data.

## Data Availability

All data generated or analysed during this study are included in this published article.

## Abbreviations

95% CI: 95% Confidence interval
ANOVA: Analysis of variance
Beta-CTx: C-telopeptides of collagen bonds
BSAP: Bone-specific alkaline phosphatase
CG: Control group
CNS: Central nervous system
EGpmc: Experimental group with passive movements with gentle joint compression
EGrlt: Experimental group with reflex locomotion therapy
GA: Gestational age
HGUE: General University Hospital of Elche
HTA: Torrecárdenas University Hospital of Almería
HUCVA: Virgen de la Arrixaca Clinical University Hospital
NICU: Neonatal intensive care unit
NTx: N-telopeptides of collagen bonds
OC: Osteocalcin
PINP: Carboxyl-terminal collagen type I propeptides
PMC: Passive movements with gentle compression
RLT: Reflex locomotion therapy

## References

[1] Litmanovitz I, Dolfin T, Regev R (2004). Bone turnover markers and bone strength during the first weeks of life in very low birth weight premature infants. J Perinat Med.

[2] Romero Barco CM, Manrique Arija S, Rodríguez PM (2012). Biochemical markers in osteoporosis: usefulness in clinical practice. Reumatol Clin.

[3] Seibel MJ (2006). Biochemical markers of bone turnover part II: clinical applications in the management of osteoporosis. Clin Biochem Rev.

[4] Ramón AM, Espuelas CF, Calmarza PC, Gracia SR, Del Cacho MJO (2017). Risk factors and biochemical markers in metabolic bone disease of premature newborns. Rev Chil Pediatr.

[5] Beyers N, Alheit B, Taljaard JF, Hall JM, Hough SF (1994). High turnover osteopenia in preterm babies. Bone.

[6] Garnero P (2017). The Utility of Biomarkers in Osteoporosis Management. Mol Diagn Ther.

[7] Fewtrell MS, Williams JE, Singhal A, Murgatroyd PR, Fuller N, Lucas A (2009). Early diet and peak bone mass: 20 year follow-up of a randomized trial of early diet in infants born preterm. Bone.

[8] Engan M, Vollsæter M, Øymar K (2019). Comparison of physical activity and body composition in a cohort of children born extremely preterm or with extremely low birth weight to matched term-born controls: a follow-up study. BMJ Paediatr open.

[9] Torró-Ferrero G, Fernández-Rego FJ, Gómez-Conesa A (2021). Physical therapy to prevent osteopenia in preterm infants: a systematic review. Child (Basel, Switzerland).

[10] Nikander R, Sievänen H, Heinonen A, Daly RM, Uusi-Rasi K, Kannus P (2010). Targeted exercise against osteoporosis: a systematic review and meta-analysis for optimising bone strength throughout life. BMC Med.

[11] Mazess RB, Whedon GD (1983). Immobilization and bone. Calcif Tissue Int.

[12] Slemenda C, Miller J (1991). Role of physical activity in the development of skeletal mass in children. J Bone Miner Res.

[13] Giannantonio C, Papacci P, Ciarniello R (2010). Chest physiotherapy in preterm infants with lung diseases. Ital J Pediatr.

[14] El-shaarawy MK, Rahman SAA, Fakher M, El A, Salah WM (2017). Effect of rolling on oxygen saturation and incubation period in preterm neonates with respiratory distress syndrome. Int J Dev Res.

[15] Sanz-Esteban I, Calvo-Lobo C, Ríos-Lago M, Álvarez-Linera J, Muñoz-García D, Rodríguez-Sanz D (2018). Mapping the human brain during a specific Vojta’s tactile input: The ipsilateral putamen’s role. J Neuroeng Rehabil.

[16] Sanz-Esteban I, Cano-de-la-cuerda R, San-Martín-Gómez A (2021). Cortical activity during sensorial tactile stimulation in healthy adults through Vojta therapy. A randomized pilot controlled trial. J Neuroeng Rehabil.

[17] Gharu RGM, Bhanu (2016). Effect of Vojta Therapy and Chest Physiotherapy on Preterm Infants with Respiratory Distress Syndrome-An Experimental Study. Indian J Physiother Occup Ther An Int J.

[18] Kole J, Metgud D (2014). Effect of lung squeeze technique and reflex rolling on oxygenation in preterm neonates with respiratory problems: A randomized controlled trial. Indian J Heal Sci.

[19] World Medical Association (2013). World Medical Association Declaration of Helsinki. JAMA.

[20] Moher D, Hopewell S, Schulz KF, CONSORT,  (2010). explanation and elaboration: updated guidelines for reporting parallel group randomised trials. BMJ.

[21] Nemet D, Dolfin T, Litmanowitz I, Shainkin-Kestenbaum R, Lis M, Eliakim A (2002). Evidence for exercise-induced bone formation in premature infants. Int J Sports Med.

[22] Eliakim A, Dolfin T, Weiss E, Shainkin-Kestenbaum R, Lis M, Nemet D (2002). The effects of exercise on body weight and circulating leptin in premature infants. J Perinatol.

[23] Massaro AN, Hammad TA, Jazzo B, Aly H (2009). Massage with kinesthetic stimulation improves weight gain in preterm infants. J Perinatol.

[24] Moyer-Mileur LJ, Ball SD, Brunstetter VL, Chan GM (2008). Maternal-administered physical activity enhances bone mineral acquisition in premature very low birth weight infants. J Perinatol.

[25] Moyer-Mileur LJ, Brunstetter V, McNaught TP, Gill G, Chan GM (2000). Daily physical activity program increases bone mineralization and growth in preterm very low birth weight infants. Pediatrics.

[26] Litmanovitz I, Dolfin T, Friedland O (2003). Early physical activity intervention prevents decrease of bone strength in very low birth weight infants. Pediatrics.

[27] Moyer-Mileur LJ, Luetkemeier M, Boomer L, Chan GM (1995). Effect of physical activity on bone mineralization in premature infants. J Pediatr.

[28] Vignochi CM, Miura E, Canani LHS (2008). Effects of motor physical therapy on bone mineralization in premature infants: a randomized controlled study. J Perinatol.

[29] Shah VS, Ohlsson A. Venepuncture versus heel lance for blood sampling in term neonates. Cochrane Database Syst Rev 2011;(10):CD001452. doi:10.1002/14651858.CD001452.pub4 10.1002/14651858.CD001452.pub4 PMC6984677 21975734

[30] Giordano V, Edobor J, Deindl P (2019). Pain and sedation scales for neonatal and pediatric patients in a preverbal stage of development: a systematic review. JAMA Pediatr.

[31] Malarvizhi G, Vatsa M, Roseline M, Nithin S, Paul S (2012). Inter-rater Reliabilty of Neonatal Infant Pain Scale NIPS as a Multidimensional Behavioral Pain Tool Nitte Univ J Heal Sci.

[32] Suraseranivongse S, Kaosaard R, Intakong P (2006). A comparison of postoperative pain scales in neonates. Br J Anaesth.

[33] Mangat A, Oei J-L, Chen K, Quah-Smith I, Schmölzer G (2018). A Review of non-pharmacological treatments for pain management in newborn infants. Children.

[34] da Motta G de CP, da Cunha MLC (2015). Prevention and non-pharmacological management of pain in newborns Rev Bras Enferm.

[35] Molina FC (2003). Marcadores Bioquímicos de Remodelado Óseo. Rev Metab oseo y Miner.

[36] Chou JH, Roumiantsev S, Singh R (2020). PediTools electronic growth chart calculators: Applications in clinical care, research, and quality improvement J Med Internet Res.

[37] Fenton TR, Kim JH (2013). A systematic review and meta-analysis to revise the Fenton growth chart for preterm infants. BMC Pediatr.

[38] Faul F, Erdfelder E, Buchner A (2009). Lang AG Statistical power analyses using G*Power 3.1: Tests for correlation and regression analyses. Behav Res Methods.

[39] Cohen J. Statistical Power Analysis for the Behavioral Sciences (2nd ed.). Routledge; 1988. 10.4324/9780203771587.

[40] R Core Team. R: A language and environment for statistical computing. 2020. https://www.r-project.org/.

[41] Cárdenas Castro JM (2014). Potencia estadística y cálculo del tamaño del efecto en G*Power: complementos a las pruebas de significación estadística y su aplicación en psicología. Salud Soc.

[42] Appelman-Dijkstra NM, Papapoulos SE (2015). Modulating Bone Resorption and Bone Formation in Opposite Directions in the Treatment of Postmenopausal Osteoporosis. Drugs.

[43] Sezer Efe Y, Erdem E, Prof TG (2019). The effect of daily exercise program on bone mineral density and cortisol level in preterm infants with very low birth weight: A randomized controlled trial. J Pediatr Nurs.

[44] Chen H-L, Lee C-L, Tseng H-I, Yang S-N, Yang R-C, Jao H-C (2010). Assisted exercise improves bone strength in very low birthweight infants by bone quantitative ultrasound. J Paediatr Child Health.

[45] Reynaga Montecinos B, Zeni SN (2009). Marcadores bioquímicos del remodelamiento óseo: Utilidad clínica. Acta bioquímica clínica Latinoam.

[46] Sharma U, Pal D, Prasad R (2014). Alkaline phosphatase: An overview. Indian J Clin Biochem.

[47] El-Farrash R, Abo-Seif I, El-Zohiery A, Hamed G, Abulfadl R (2019). Passive Range-of-Motion Exercise and Bone Mineralization in Preterm Infants: A Randomized Controlled Trial. Am J Perinatol.

[48] Vignochi CM, Silveira RC, Miura E, Canani LHS, Procianoy RS (2012). Physical therapy reduces bone resorption and increases bone formation in preterm infants. Am J Perinatol.

[49] Aita M, De C-F, Lavallée A (2020). Effectiveness of Interventions on early neurodevelopment of preterm infants: a systematic review and meta-analysis. BMC Pediatr.

[50] Pados BF, McGlothen-Bell K (2019). Benefits of infant massage for infants and parents in the NICU. Nurs Womens Health.

